# FMRpolyG-positive inclusions in CNS and non-CNS organs of a fragile X premutation carrier with fragile X-associated tremor/ataxia syndrome

**DOI:** 10.1186/s40478-014-0162-2

**Published:** 2014-11-26

**Authors:** Ronald AM Buijsen, Chantal Sellier, Lies-Anne WFM Severijnen, Mustapha Oulad-Abdelghani, Rob FM Verhagen, Robert F Berman, Nicolas Charlet-Berguerand, Rob Willemsen, Renate K Hukema

**Affiliations:** Department of Clinical Genetics, Erasmus, MC PO Box 2040, 3000CA The Netherlands; Department of Neurobiology and Genetics, IGBMC, INSERM U964, CNRS UMR7104, University of Strasbourg, Illkirch, France; Department of Neurological Surgery, UC Davis, Davis, CA 95618 USA

**Keywords:** FXTAS, CGG repeat, FMRpolyG, RAN translation, Gain-of-function, Inclusions

## Abstract

para

**Electronic supplementary material:**

The online version of this article (doi:10.1186/s40478-014-0162-2) contains supplementary material, which is available to authorized users.

Fragile X-associated Tremor/Ataxia syndrome (FXTAS), a late-onset monogenetic neurodegenerative disorder, is caused by a CGG-repeat expansion (55-200) in the 5′ UTR of the fragile-X mental retardation 1 gene (*FMR1*) on the X-chromosome [1]. The prevalence of the *FMR1* premutation (PM) is about 1:855 in males and 1:291 in females [2]. Approximately 45.5% of male and 16.5% of female PM carriers older than 50 years will develop signs of FXTAS [3]. In addition to the core features of tremor and gait ataxia, unexplained medical co-morbidities have been reported, including thyroid disease, cardiac arrhythmias, hypertension, migraine, impotence, and neuropathy [4]. PM carriers have increased levels of *FMR1* mRNA (2 to 8 fold in leucocytes) and normal to slightly reduced FMR1 protein (FMRP) levels [5]. The current hypothesis is that FXTAS is caused by an RNA gain-of-function mechanism. Ubiquitin-positive intranuclear inclusions, are found in both brain and non-central nervous system (CNS) organs of patients with FXTAS [6,7]. So far, it is not clear whether these inclusions are protective or toxic. Recently, it has been hypothesized that repeat-associated non-AUG (RAN) translation plays a role in disease process and inclusion formation. Todd et al. [8] demonstrated that through initiation at a near-ATG codon located in the 5′UTR of the *FMR1* gene a polyGlycine-containing protein, FMRpolyG, is expressed. This protein accumulates in ubiquitin-positive inclusions in *Drosophila*, cell culture, mouse disease models and brain from FXTAS patients. To investigate the link between FMRpolyG expression and the co-morbid medical problems associated with the PM we have developed two novel mouse monoclonal antibodies against polyGlycine; 8FM and 9FM (for epitopes and specificity see Additional file 1: Figure S1), and performed immunostaining in CNS as well as in non-CNS organs of FXTAS patient J.L. (case 6 in [7]; other cases not available). To establish antibody specificity, we performed immunostaining with both antibodies on brain sections from FXTAS patient J.L., healthy non-demented controls (n = 3) and a patient with Parkinson disease, Alzheimer disease, or C9FTD. In hippocampus and cerebellum from FXTAS patient J.L. we identified FMRpolyG-positive inclusions with both 8FM (1:10) and 9FM (1:10) antibody (Figure 1a-b, Additional file 2: Figure S2a-b), as was described previously [8]. None of the controls showed FMRpolyG-positive inclusions (data not shown). Next, we studied the immunolocalization of FMRpolyG protein in heart, kidney, adrenal gland and thyroid in patient J.L. with 8FM (1:10) and 9FM (1:10), compared to post mortem non-CNS somatic organ tissues from 3 healthy controls. We also examined tissues for FMRP (mouse T1A; 1:200) expression and ubiquitin-positive inclusions (DAKO, ZO458; 1:200). Consistent with our previous report [7], ubiquitin-positive intranuclear inclusions were identified along with a normal distribution of FMRP (data not shown). Intranuclear FMRpolyG-positive inclusions could be detected in all organs examined (Figure 1c-h, Additional file 2: Figure S2c-h). No control tissues showed any FMRpolyG-positive inclusions (data not shown). Colocalization of ubiquitin- and FMRpolyG-positive inclusions was visualized and quantified by immunofluorescent double staining using antibodies against ubiquitin and FMRpolyG (8FM) (Figure 2a-f). For hippocampus, cerebellum and the non-CNS organs most inclusions are positive for both FMRpolyG and ubiquitin, although some rare inclusions positive for only one of the proteins could also be detected (Figure 2g, n = 100 inclusions). In conclusion, using two novel antibodies the present report not only confirms the existence of FMRpolyG-positive aggregates in CNS tissue from a FXTAS individual but also demonstrates for the first time the presence of FMRpolyG-positive intranuclear inclusions in post mortem non-CNS material of a PM carrier with FXTAS. Furthermore, colocalization of FMRpolyG and ubiquitin is found in the vast majority of inclusions. The presence of FMRpolyG-positive intranuclear inclusions in heart, kidney, adrenal gland and thyroid is consistent with the unexplained medical co-morbidities reported in some patients with FXTAS, including thyroid disease, cardiac arrhythmias, hypertension, migraine, impotence, and neuropathy. We hypothesize that the underlying pathological mechanisms of the medical co-morbidities in systemic tissues share common features (protein toxic gain-of-function) with CNS pathology of patients with FXTAS. Our report suggests that in addition to elevated levels of *FMR1* mRNA containing an expanded CGG repeat, and ubiquitin-positive inclusions, FMRpolyG expression might also play a role in a toxic gain-of-function mechanism in medical co-morbidities in FXTAS (RNA versus FMRpolyG toxic gain-of-function). Interestingly, a very recent report suggests that RAN translation products in C9FTD/ALS, toxic dipeptide repeat proteins (poly-(glycine-arginine) and poly-(proline-arginine)), are toxic in *Drosophila* [9]. Further research is needed to understand how FMRpolyG may elicit toxicity in both CNS and non-CNS organs and its precise role in co-morbidities in PM carriers. Importantly, if FMRpolyG production is important for cellular toxicity this will open new avenues for therapeutic intervention studies for FXTAS by developing drugs that block this aberrant translation.

**Figure 1:**
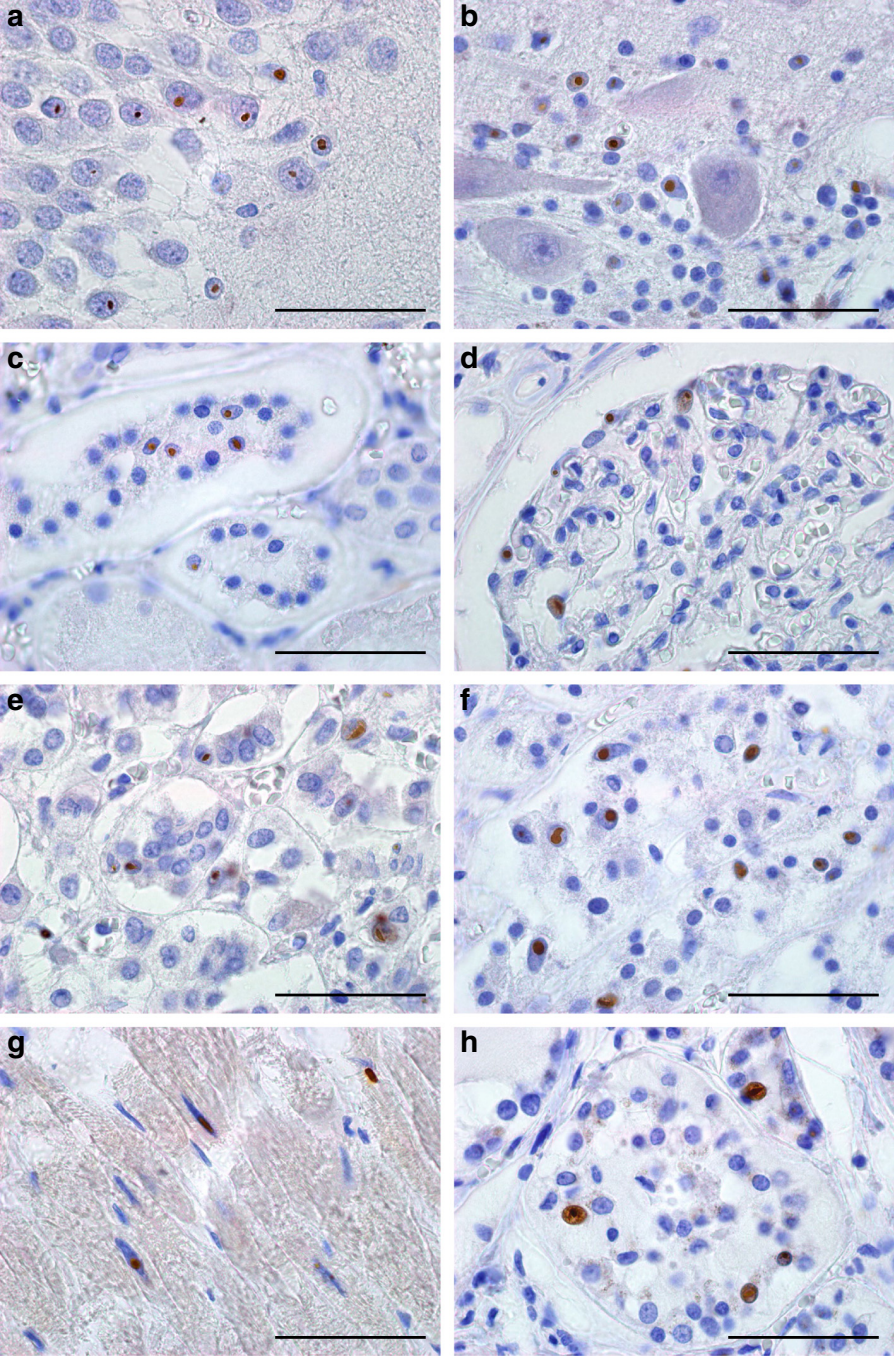
**9FM FMRpolyG-positive intranuclear inclusions in hippocampus, cerebellum and non-CNS tissues of a FXTAS patient.** FMRpolyG-positive (9FM) intranuclear inclusions in **a** hippocampus**, b** cerebellum, **c** glomeruli and **d** distal tubule of the kidney, **e** zona glomerulosa and **f** zona reticularis of adrenal gland, **g** cardiomyocytes and **h** thyroid. All sections were immunostained with 9FM antibody and counterstained with hematoxylin. Scale bars represent 50 μm.

**Figure 2:**
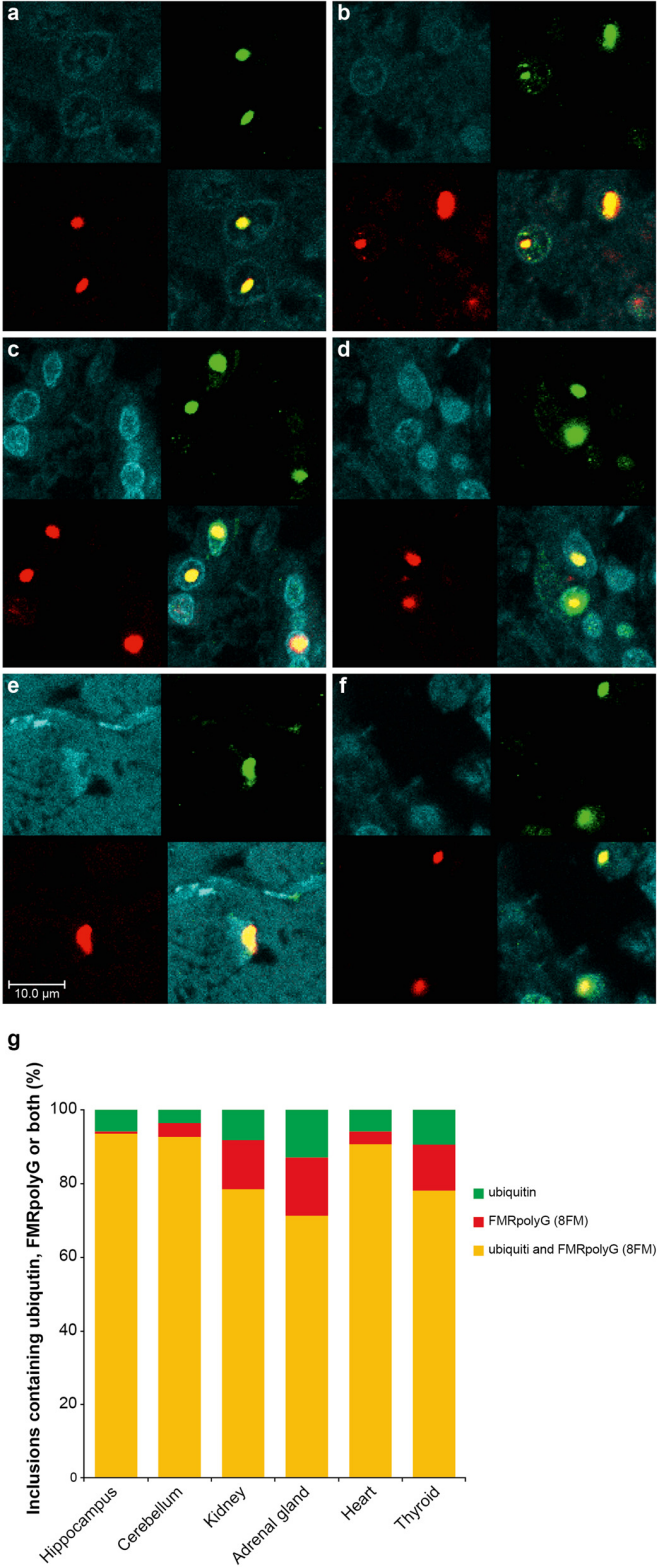
**Colocalization of FMRpolyG (8FM) and ubiquitin in intranuclear inclusions in hippocampus, cerebellum and of non-CNS tissues of a FXTAS patient.** Staining for ubiquitin (green), FMRpolyG (8FM; red) and DAPI (blue). Colocalization of ubiquitin and FMRpolyG (yellow) is seen in **a** hippocampus**, b** cerebellum, **c** kidney, **d** adrenal gland, **e** cardiomyocytes, and **f** thyroid; **g** quantification of inclusions containing ubiquitin and/or FMRpolyG (n = 100). Scale bars represent 10 μm.

## Additional files

Additional file 1: Figure S1. Epitopes and specificity FMRpolyG antibodies 8FM and 9FM. a Sequence of the 5′UTR of human *FMR1* gene and the FMRpolyG peptide sequence resulting from RAN translation. Epitopes of 8FM and 9FM antibodies are boxed. b Monoclonal 8FM and 9FM antibodies were validated on COS7 cells transfected with pEGFP (Clonetech) plasmid containing the 5′UTR of *FMR1* with 5O CGG repeats, thus expressing the polyGlycine protein fused to GFP. On Western Blot a specific product could be detected for both antibodies and no product was detectable in COS7 cells transfected with a control GFP plasmid. c As an additional control experiment we performed immunostainings for 8FM antibody and could demonstrate specific intranuclear inclusions in COS7 cells transfected with a construct expressing the FMRpolyG fused to GFP, while cells tranfected with only GFP did not show any inclusion formation. Identical results were obtained with 9FM antibody (data not shown). Additional file 2: Figure S2. 8FM FMRpolyG-positive intranuclear inclusions in hippocampus, cerebellum and non-CNS tissues of a FXTAS patient. FMRpolyG-positive (8FM) intranuclear inclusions in a hippocampus, b cerebellum, c glomeruli and d distal tubule of the kidney, e zona glomerulosa and f zona reticularis of adrenal gland, g cardiomyocytes and h thyroid. All sections were immunostained with 8FM antibody and counterstained with hematoxylin. Scale bars represent 50 μm.

## Abbreviations

ALS: Amyotrophic lateral sclerosis
C9FTD: Chromosome 9 open reading frame 72 frontotemporal dementia
CNS: Central nervous system
*FMR1*: Fragile-X mental retardation 1
FMRP: Fragile-X mental retardation 1 protein
FXTAS: Fragile X-associated Tremor/Ataxia syndrome
PM: Fragile-X mental retardation 1 premutation
RAN: Repeat-associated non-AUG
