# Supplementary figures and images for: FMRpolyG-positive inclusions in CNS and non-CNS organs of a fragile X premutation carrier with fragile X-associated tremor/ataxia syndrome
Source: Acta Neuropathol Commun. 2014 Nov 26;2:162. doi: 10.1186/s40478-014-0162-2 (PMC4254384; doi:10.1186/s40478-014-0162-2)

**a**

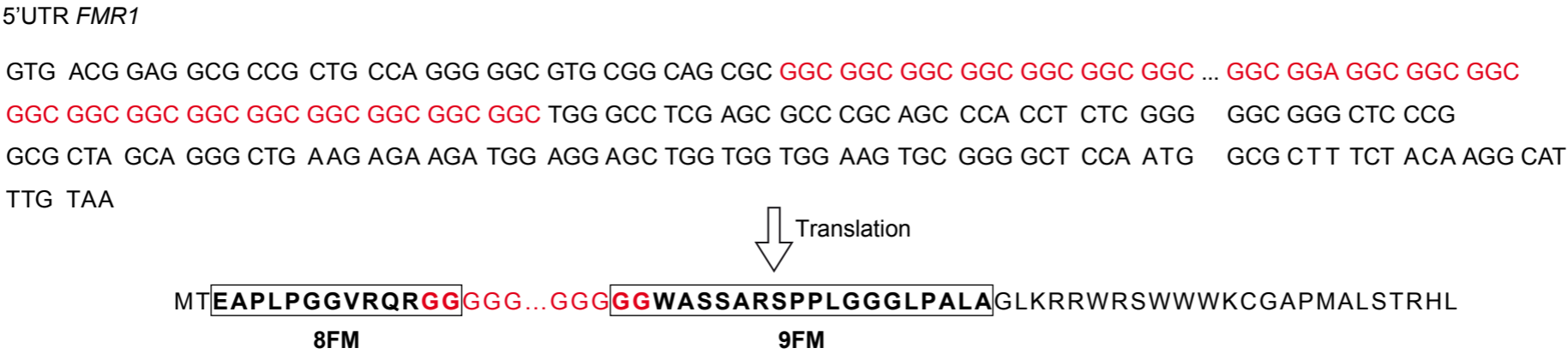

**b**

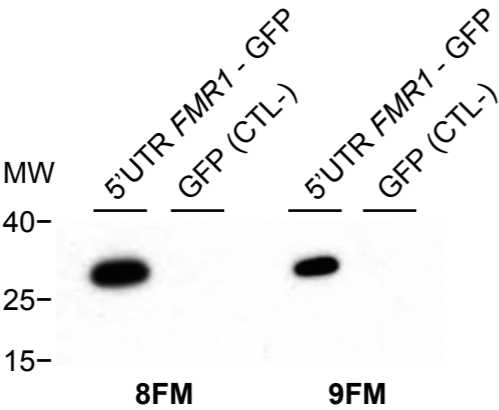

**c**

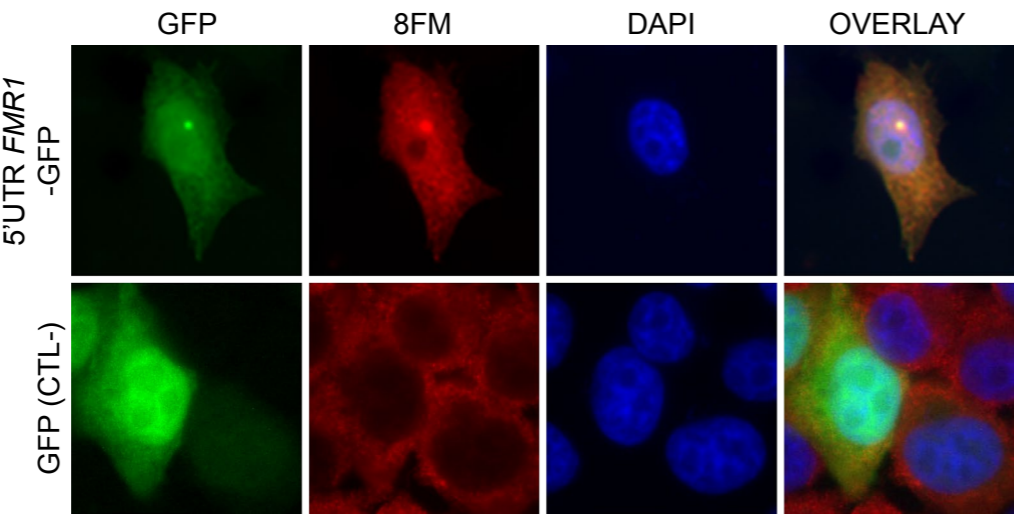

Supplement: Additional file 1: Figure S1. — Epitopes and specificity FMRpolyG antibodies 8FM and 9FM. a Sequence of the 5′UTR of human FMR1 gene and the FMRpolyG peptide sequence resulting from RAN translation. Epitopes of 8FM and 9FM antibodies are boxed. b Monoclonal 8FM and 9FM antibodies were validated on COS7 cells transfected with pEGFP (Clonetech) plasmid containing the 5′UTR of FMR1 with 5O CGG repeats, thus expressing the polyGlycine protein fused to GFP. On Western Blot a specific product could be detected for both antibodies and no product was detectable in COS7 cells transfected with a control GFP plasmid. c As an additional control experiment we performed immunostainings for 8FM antibody and could demonstrate specific intranuclear inclusions in COS7 cells transfected with a construct expressing the FMRpolyG fused to GFP, while cells tranfected with only GFP did not show any inclusion formation. Identical results were obtained with 9FM antibody (data not shown). [file 40478_2014_162_MOESM1_ESM.pdf]

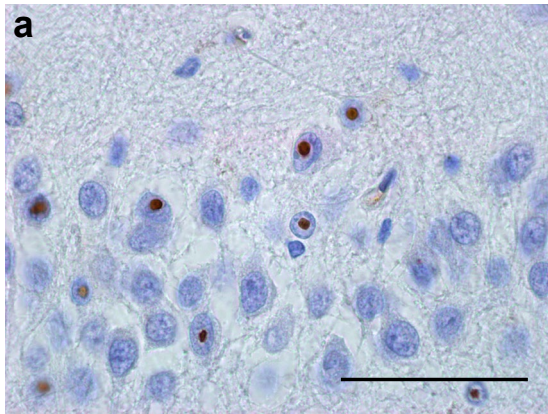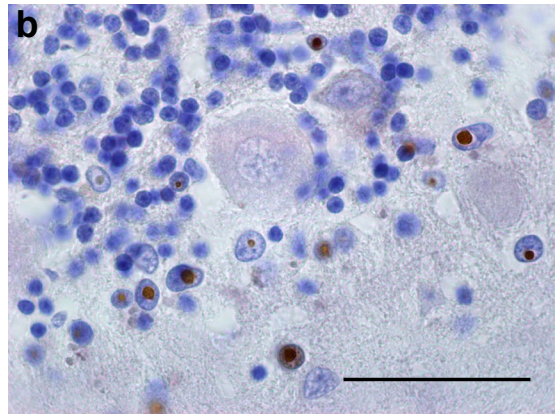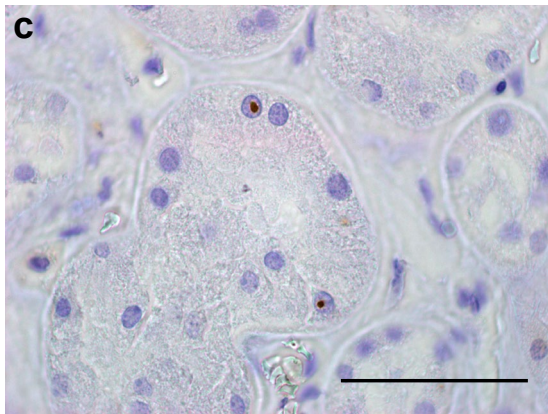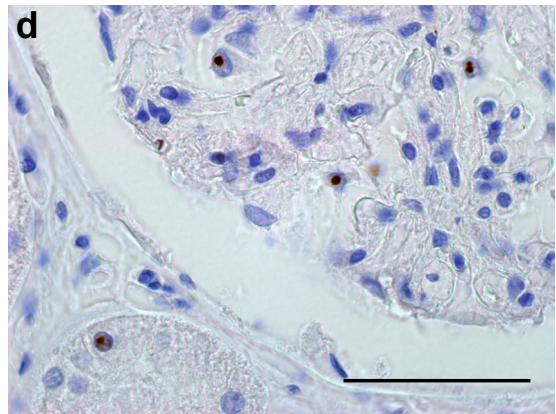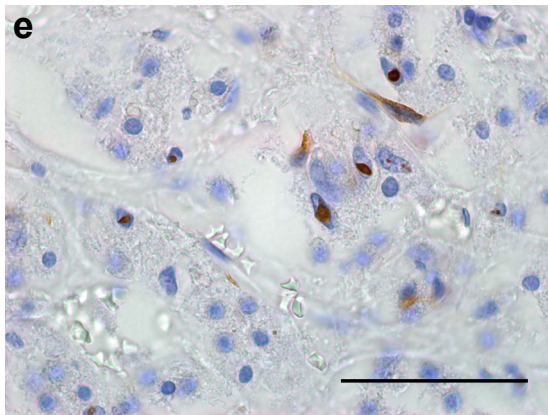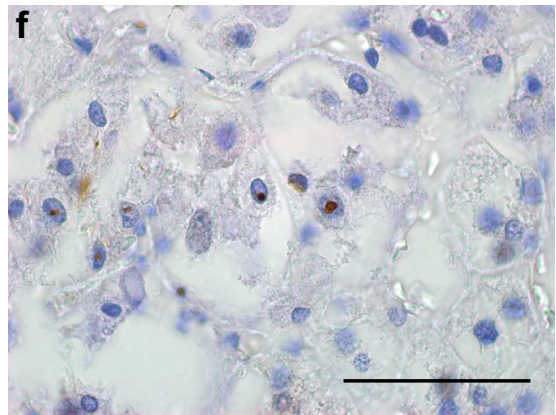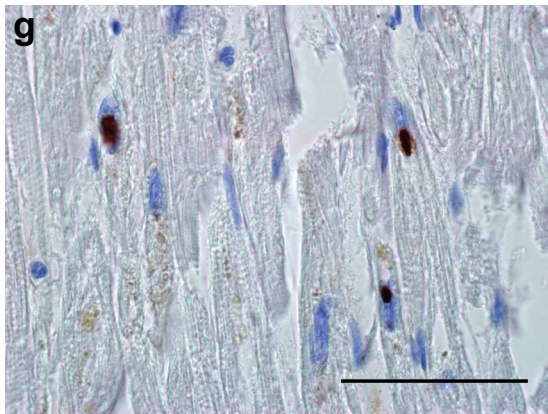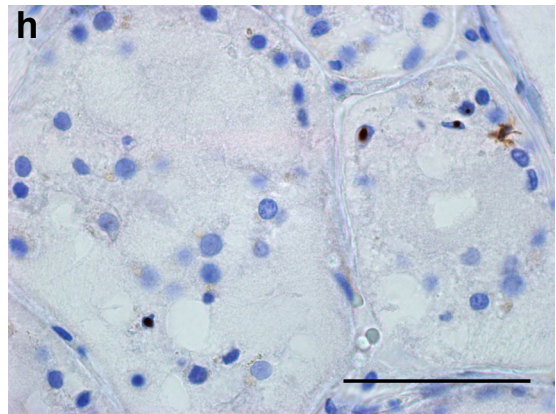

Supplement: Additional file 2: Figure S2. — 8FM FMRpolyG-positive intranuclear inclusions in hippocampus, cerebellum and non-CNS tissues of a FXTAS patient. FMRpolyG-positive (8FM) intranuclear inclusions in a hippocampus, b cerebellum, c glomeruli and d distal tubule of the kidney, e zona glomerulosa and f zona reticularis of adrenal gland, g cardiomyocytes and h thyroid. All sections were immunostained with 8FM antibody and counterstained with hematoxylin. Scale bars represent 50 μm. [file 40478_2014_162_MOESM2_ESM.pdf]
